# Indian research on disaster and mental health

**DOI:** 10.4103/0019-5545.69254

**Published:** 2010-01

**Authors:** Nilamadhab Kar

**Affiliations:** Department of Psychiatry, Mental Health Directorate, Wolverhampton City PCT, Wolverhampton, UK

**Keywords:** Disaster, India, Mental health, Research

## Abstract

The primary source for this annotation on disaster mental health research is the Indian Journal of Psychiatry. Key words like disasters, earthquake, cyclone, tsunami and flood were searched from its electronic database and relevant articles are discussed. The cross-referenced articles and relevant researches conducted on disasters in India which are published elsewhere were the secondary sources of information. There have been many epidemiological studies and only a few interventional studies on disasters in India. Prevalence figures of psychiatric disorders varied considerably across studies, secondary to nature and severity of disaster, degree of loss, support available and probably also due to the study methodology. Suggestions for intervention included pre-disaster planning, training of disaster workers, utilization of community-level volunteers as counselors, and strengthening existing individual, social and spiritual coping strategies. There is a need for more longitudinal follow-up studies and interventional studies.

## INTRODUCTION

A variety of articles on post-disaster psychiatric research has been published in the Indian Journal of Psychiatry. The relevance of mental health issues following disasters has also been highlighted in many presidential addresses and editorials in recent years.[1–6] It has been acknowledged that India has been traditionally vulnerable to natural disasters on account of its unique geo-climatic conditions. Both natural and manmade disasters occur quite regularly in India, like many in the developing world.[2,5,7] About 60% of the Indian landmass is prone to earthquakes of various intensities; over 40 million hectares are prone to floods; about 8% of the total area is prone to cyclones and 68% of the areas are susceptible to drought.[2]

Recent major natural disasters were Marathwada earth quake (1993), Andhra Pradesh cyclone (1996), Jabalpur earthquake (1997), super cyclone in Orissa (1999), Gujarat earthquake (2001) and tsunami in Tamil Nadu (2004). There have been major industrial accidents like Bhopal Gas tragedy (1984); the effects of which are still being felt. Manmade disasters are common too, like terrorism, communal riots and violence. Recently, the bomb blasts in many Indian cities and terror attack in Mumbai have been extremely traumatic.

The human and economic cost of frequent disasters in India has been colossal.[1,5] It has been highlighted that there is an adverse impact on the mental health of not only the surviving victims but also their relatives, rescuers and disaster workers. Victim groups who are specifically at risk have been identified, e.g. children and adolescents, elderly, those with past psychiatric history etc.[1,3,8]

## EPIDEMIOLOGICAL STUDIES

### Natural disasters

A study conducted a month after the Latur earthquake found psychiatric morbidity in 59%; posttraumatic stress disorder (PTSD) in 23% and major depression in 21% being the common diagnoses.[9] Another study following Latur earthquake in Marathwada revealed that survivors had PTSD (74%), major depression (89%), generalized anxiety disorder (GAD) (42%) and panic disorder (28%).[1] An Indian Council of Medical Research (ICMR) study in Latur found that 21.5% of adult males in the affected group received a psychiatric diagnosis compared to 13.1% in the controls; corresponding figures for adult females were 14.9% and 5.1% respectively.[10]

The psychiatric sequelae of the Orissa super-cyclone in 1999 suggested that 80.4% of the subjects had probable psychiatric disorder. PTSD was found in 44.3%; anxiety disorder in 57.5% and depression in 52.7%. A considerable proportion (63.4%) of victims with psychiatric disorder had comorbidity. Children and adolescents, elderly persons, lower socioeconomic status (SES), lower educational levels, unemployment, physical injury, degree of exposure, need for evacuation, death in the family, fear of imminent death during the event, hopelessness, increased stress before disaster and past psychiatric history were associated with adverse psychological sequelae. Increase in suicidality was observed.[8]

Around one year after the super-cyclone in Orissa, a study on adolescents found that the prevalence of PTSD was 26.9%, depression 17.6%, and GAD 12.0%. Proportion of adolescents with any diagnosis was 37.9%. Comorbidity was found in 39.0% of adolescents with a psychiatric diagnosis. Adolescents from middle SES were more affected. Prolonged periods of helplessness and lack of adequate post-disaster psychological support were perceived as probable influencing factors other than the severity of the disaster.[11] Another study on children following the super-cyclone, found that PTSD presentations were similar to that in other cultures. It was felt that, though highly prevalent, PTSD might be missed without clinical screening. PTSD was present in 30.6%, and an additional 13.6% had sub-syndromal PTSD. Parents or teachers reported mental health concerns in 7.2% subjects, who were only a minor proportion (12.8%) of subjects with any syndromal diagnosis. Significantly more (43.7%) children in high-exposure areas had PTSD than those (11.2%) in low-exposure areas. Depression was significantly associated with PTSD. High exposure, lower educational level and middle SES significantly predicted PTSD. Extreme fear and perceived threat to life during disaster, death in family, damage to home, stay in shelters were not significantly associated with PTSD in children.[12]

The prevalence rates for psychiatric disorders (27.2%) and psychological symptoms (79.7%) around six to nine months following the tsunami in coastal Tamil Nadu have been considerable. The commonest psychiatric disorder was depression, followed by alcohol use disorders in males and anxiety disorders in females. The rate of PTSD, 12.5/1000, was found to be lower than expected. It was perceived that the psychological symptoms get taken care of by the informal social mechanism and counselors working with non-governmental organizations (NGO) and that the specialist psychiatric services are required for a smaller proportion of populations.[13]

In a study of tsunami-affected males in Kanyakumari, 43% had clinically significant psychological distress, and 31% had very high levels of psychological distress. Individuals with higher frequency of personal prayer, better quality of marital life, job satisfaction were relatively protected; whereas substance abuse and severe disaster experience such as losing a family member were risk factors for severe psychological distress.[14]

Initial assessment in the Andaman and Nicobar Islands during the early phase of the 2004 tsunami disaster revealed 5-8% of the population was suffering from significant mental health problems. The authors expected that the psychiatric morbidity would be around 25-30% in the disillusionment phase. High resilience was seen in the joint family system of the tribal Nicobarese.[15] Psychiatric morbidity in these islands during the first three months following the earthquake and tsunami was significantly more (5.2%) in the displaced population than (2.8%) the non-displaced. The overall psychiatric morbidity was 3.7%. The disorders included panic disorder, unspecified anxiety disorder, and somatic complaints. The existence of an adjustment disorder was significantly higher in the non-displaced survivors. Depression and PTSD were distributed equally in both groups.[16]

The most common psychiatric morbidities in children and adolescents as primary (exposed directly to tsunami and earthquake) and secondary (those with close family and personal ties with primary survivors) survivors in the Andaman and Nicobar were adjustment disorder (13.5%), depression (13.5%), panic disorder (10.8%), PTSD (10.8%), schizophrenia (2.7%), and other disorders (43.2%). Sub-clinical syndrome was present in the majority of the primary and secondary survivors. A majority of survivors required community-based group interventions.[17]

Fire disasters are common in India. A study in a Delhi slum following a fire disaster reported that the prevalence of psychiatric disorders was significantly higher (7.8%) compared to that (2.2%) in the control group; prevalence of psychological ill health was also higher (23.2% vs. 5.0% respectively). The common psychiatric disorders were depression, substance use disorders, GAD and somatoform disorder. Age and participation in relief work were found to be strong predictors and physical injuries were found to be a weak predictor of mental health morbidity.[10]

Following a fire disaster in Bangalore in 1981, 35.8% of the bereaved relatives had psychiatric symptoms requiring treatment.[10,18] In another study, following Mandi Dabwali fire disaster 56% of children had PTSD after two months.[19]

### Industrial disasters

A study conducted within three months of the Bhopal gas tragedy yielded a 22.6% prevalence of mental disorders. Most of the patients were females (81.1%), and under 45 years of age (74%). The main diagnoses were anxiety neurosis (25%), depression (20%) and adjustment reaction with predominant disturbance of emotions (16%). Cases of psychosis were rare.[1,20]

In a community-based study of a representative sample of the gas-exposed population, the prevalence of psychiatric morbidity in the exposed population after one and a half years of disaster was found to be significantly higher in comparison to control group (9.4% vs. 2.5%).[10,21]

### Manmade disasters

Following the Mumbai riots in 1992-93 a study on hospital ized victims found them in a state of shock, fear and helplessness; 33% expressed anger; 2% of these (all female who saw the mangled bodies of their husbands) had attempted suicide; 21% of those interviewed had severe anxiety, 41% had paranoid thinking and obsessional symptoms and majority had loss of libido. PTSD features scored very high; and a few were emotionally dumb; and 36% had suicidal thoughts.[1,22]

Within days of a bomb blast (1996) in a bus in a terrorist activity in Dausa, Rajasthan 35.5% had psychiatric morbidity: 19.4% had acute stress reaction, 9.7% had depression and 6.5% dissociative amnesia. The most commonly reported symptoms were depersonalization, derealization, sleep disturbances, specially generalized sleep loss, loss of appetite, nightmares, situational anxiety, depression, mental irritability, dullness of feelings, self blame, guilt, loss of interest, suicidal ideas, and worry about money, spouse, work and children.[23]

Four years after exposure to communal violence in Ahmedabad PTSD was found in 4.7% of children and adolescents; and 9.4% had major depression. PTSD was associated with age older than 12 and residence in Ahmedabad, the worst affected city; it was not associated with gender, religion, change of residence, income or education.[24]

### Other studies

A study explored the experiences of women who were traumatized by the communal riots in Ahmedabad, in 2002. Victims described experiences that closely resembled re-experiencing, avoidance and hyperarousal. The authors concluded that PTSD may be a relevant clinical construct in the Indian context.[25]

The challenges in diagnosing PTSD in children have been highlighted in a communiqué observing the differences in trauma responses in children compared to adults, paucity of literature on these from developing countries, complicating post-disaster situations with grief reactions, survivor guilt and trauma-induced demoralization and associated comorbidities. The authors suggested use of semistructured interview schedules, self/parent report instruments, play and projective psychological tests, spending adequate time with children to elicit symptoms of PTSD.[26]

A study looked into neurocognitive function in methyl isocynate (MIC)-exposed women who were affected by the Bhopal gas disaster and compared it with those of normal control using PGI-Battery of Brain dysfunctions. The result suggested that the MIC-exposed women had significant neurocognitive dys functions compared to controls in immediate recall, visual retention, and in results of Nahar-Bensen and Bender-Gestalt tests.[27]

### Post-disaster Interventions

Mental healthcare for the disaster-affected populations is viewed in the domain of public mental health whose importance is growing in India.[6] The role of psychiatrists in disaster management has been stressed.[4]

There has been a national initiative for disaster management; however, the need for involvement of mental health professionals in these has not been highlighted; indeed crisis management and psychosocial care have not been adequately recognized in the mainstream disaster management work. The poverty of administrative response to include mental healthcare in disaster management planning and work has also been mentioned.[1] Governments everywhere measure the magnitude of disasters by estimating loss in terms of lives and money. Relief agencies are mainly concerned with providing for physical needs and attending to physical injuries. It has been stressed that the emotional injuries also need caring otherwise they can predispose a large number of victims of disasters to long-term mental health sequelae.[7] The Indian Psychiatric Society has a taskforce on disaster management,[2] which is best placed to influence the national strategy to include mental healthcare for disaster victims.

Many authors in the Indian Journal of Psychiatry have suggested interventional methods for the disaster victims. Highlighted are the concepts like prevention of psychological disorders, preparedness, organization of mental health teams at disaster sites, prioritizing care for the groups with higher risk, rehabilitation, involvement of local individuals and organizations in post-disaster psychosocial work and training the primary care health professionals to provide mental healthcare.[1,28] It has been emphasized that disaster-related mental health issues need phase-appropriate responses and interventions, considering the five conceptual phases, namely: pre-disaster warning phase; disaster phase: during and immediately after the disaster; early; recent; and remote post-disaster phases.[29,30]

In an interesting article published in 1963, psychiatric ‘First Aid’ in community disasters has been discussed with specific reference to nuclear warfare.[31] It is noted that ‘human kindness and generosity are seen in abundance towards those afflicted by any disasters, but rarely is the mind of the injured person considered from the scientific and professional aspects when first aid is thought of and administered’. It was predicted that more than 50% of the large number of casualties of war will be psychiatric in nature. The article emphasized the need for psychiatric First Aid to deal with these cases effectively. It has described various effects of war trauma from normal reactions to panic, depressed, hyperactive, somatic, psychotic and delayed reactions and the nature of First Aid for these. It briefly mentions the predisposing factors and mechanisms for ‘breakdown’.

Suggested methods of First Aid included: prompt and firm support, personal attention to make the victim feel less desolate, quiet supervision, suggestion to carry out simple routine tasks of helping others less fortunate than themselves, simple explanations, reassurance, explanation to make the victim understand that regardless of the cause of the disaster the damage must be repaired by coordinated effort by all available personnel. For psychotic reactions, the author mentioned that these were usually short-lived being known as ‘three day psychosis’ which might disappear with removal from the traumatic situations, however, sometimes sedation and Electroconvulsive therapy (ECT) might be advisable, if other methods did not work.[31]

Psychological First Aid has also been stressed in a dedicated editorial on the mental health perspective of disaster.[5] It describes debriefing and defusing, crisis reduction counseling, crisis intervention in post-disaster situation. This article also suggests preparing the disaster personnel in crisis intervention and emergency management during the pre-disaster planning. Factors influencing recovery have been highlighted.

In India a wide range of non-specialist personnel as well as volunteers discharge a wide variety of mental healthcare tasks including those following disasters.[28] It has been suggested that the disaster mental health teams must be able to understand the local culture, traditions, language, belief systems and local livelihood patterns to respond to a high-magnitude disaster. They also need to integrate with the network of various relief agencies to cater to the needs of the survivors. The presence of a disaster mental health team is considered as a definite requirement during the early phase of the disaster in developing countries.[15] It has been reported that community-based group interventions are simple, easy to implement using local resources, effective in all groups and provide important components of psychosocial rehabilitation. There are many examples of this kind of intervention following disasters, in the Indian setup.[8,17,32]

An intervention program for children a year after tsunami found that, only hyperactivity problems were significantly reduced after intervention. Children in the intervention group appreciated expression of positive emotions and were also more likely to desist from smoking compared to the control group. The majority of the children were likely to be resilient and only children with preexisting vulnerability required specific and specialized interventions.[33]

A qualitative study through focus group discussions nine months after the tsunami in Tamil Nadu reported that participants reconstructed meaning for the causes and the aftermath of the disaster in their cultural idiom. Qualitative changes in their social structure, processes and attitudes towards different aspects of life were revealed. Survivors valued their unique individual, social and spiritual coping strategies more than formal mental health services. The results of this study suggested that interventions after disaster should be grounded in ethno-cultural beliefs and practices and should be aimed at strengthening prevailing community coping strategies.[34]

An experiential account of mental healthcare following the devastating earthquake of Latur is worth mentioning. A mental health camp was set up in a temple by a group of psychiatrists. The author writes, “We had just begun our ‘funda’ with our PTSD business, when the villagers cracked a joke on their own. As they continued, the session became hilarious and we fulfilled our agenda. The fantastic component was that the villagers set the agenda and the method of healing through their own cultural and mental processes.” This observation highlights many important aspects of mental healthcare in a disaster-affected community. He reflects, “The mantra for disaster worker is that when a survivor initiates laughter, go with it but do not jump on the bandwagon and be a driver. Let him/her be and you gently nudge the process with all participants as partners in the process”.[35] The above reaffirmed the healing elements of humor; however, being able to utilize it in post-disaster situations could be a tough task.

There are a few other researches and observations on disasters in India published elsewhere which can be used as additional sources of information on this subject.[36,37,38,39,40]

## CONCLUSIONS

There is a great need for long-term prospective studies on the effects of disaster and more interventional studies to find out the effectiveness of supportive measures provided to the victims. Factors that can prevent psychiatric morbidity in the survivors need to be ascertained. It is imperative to inculcate a mental health support system in the disaster response strategies in India.

## References

[1] Kar GC (2000). Disaster and mental health. Indian J Psychiatry.

[2] Mohandas E (2009). Roadmap to Indian Psychiatry. Indian J Psychiatry.

[3] Shastri PC (2009). Promotion and prevention in child mental health. Indian J Psychiatry.

[4] Reddy IR (2007). Making psychiatry a household word. Indian J Psychiatry.

[5] Rao TSS (2004). Managing impact of natural disasters: some mental health issues. Indian J Psychiatry.

[6] Desai NG (2006). Public mental health: An evolving imperative. Indian J Psychiatry.

[7] Khandelwal SK (2006). The joy of mental health: Some popular writings of Dr. NN Wig Book Review. Indian J Psychiatry.

[8] Kar N, Jagadisha T, Sharma PS, Murali N, Mehrotra S (2004). Mental health consequences of the trauma of supercyclone 1999 in Orissa. Indian J Psychiatry.

[9] Sharan P, Chaudhary G, Kavathekar SA, Saxena S (1996). Preliminary report of psychiatric disorders in survivors of a severe earthquake. Am J Psychiatry.

[10] Desai NG, Gupta DK, Srivastava RK (2004). Prevalence, pattern and predictors of mental health morbidity following an intermediate disaster in an urban slum in Delhi: A modified cohort study. Indian J Psychiatry.

[11] Kar N, Bastia BK (2006). Post-traumatic stress disorder, depression and generalised anxiety disorder in adolescents after a natural disaster: A study of comorbidity. Clin Pract Epidemiol Mental Health.

[12] Kar N, Mohapatra PK, Nayak KC, Pattnaik P, Swain SP, Kar HC (2007). Post-traumatic stress disorder in children and adolescents one year after a super-cyclone in Orissa, India: Exploring cross-cultural validity and vulnerability factors. BMC Psychiatry.

[13] Nambi S, Desai NG, Shah S (2007). Mental health morbidity and service needs in tsunami affected population in coastal Tamil Nadu. Indian J Psychiatry.

[14] George C, Sunny G, John J (2007). Disaster experience, substance abuse, social factors and severe psychological distress in male survivors of the 2004 tsunami in South India. Indian J Psychiatry.

[15] Math SB, Girimaji SC, Benegal V, Uday Kumar GS, Hamza A, Nagaraja D (2006). Tsunami: Psychosocial aspects of Andaman and Nicobar islands. Assessments and intervention in the early phase. Int Rev Psychiatry.

[16] Math SB, John JP, Girimaji SC, Benegal V, Sunny B, Krishnakanth K (2008). Comparative study of psychiatric morbidity among the displaced and non-displaced populations in the Andaman and Nicobar Islands following the tsunami. Prehosp Disaster Med.

[17] Math SB, Tandon S, Girimaji SC, Benegal V, Kumar U, Hamza A (2008). Psychological impact of the tsunami on children and adolescents from the andaman and nicobar islands: Prim Care Companion. J Clin Psychiatry.

[18] Narayanan HS, Sathyabati K, Nardev G, Thakar S (1987). Grief reaction among bereaved relatives following a fire disaster in a circus. NIMHANS J.

[19] Sharma A, Rao S, Srinivas S (1998). Mandi Dabwali fire disaster. Psychological impact on school children after two months-preliminary findings. Indian J Psychiatry.

[20] Murthy RS (1997). Psychological consequences of Bhopal disaster. In: Proceedings of National workshop on psychosocial consequences of disasters.

[21] Bhiman A (2001). Mental health consequences of Bhopal gas tragedy. Paper presented in the 17^th^ World Congress of Social Psychiatry, Agra: 27-31 October.

[22] Shetty H, Chhabria A (1997). Bombay riots. In Proceedings of National workshop on Psychosocial consequences of disasters.

[23] Gautam S, Gupta ID, Batra L, Sharma H, Khandelwal R, Pant A (1998). Psychiatric morbidity among victims of bomb blast. Indian J Psychiatry.

[24] Vankar GK, Banwari G, Parikh V, Shah H (2007). PTSD in children and adolescents: Four years after the communal violence. Indian J Psychiatry.

[25] Mehta K, Vankar G, Patel V (2005). Validity of the construct of post-traumatic stress disorder in a low-income country: Interview study of women in Gujarat, India. Br J Psychiatry.

[26] Murali N, Kar N, Jagadisha (2002). Recognition and clinical assessment of childhood PTSD. Indian J Psychiatry.

[27] Sahu RN, Naik GP, Dusad A, Agrawal VK (2005). Neurocognitive function in women affected by the Bhopal gas disaster. Indian J Psychiatry.

[28] Murthy RS (2009). Introduction to psychiatry: Book Review. Indian J Psychiatry.

[29] Kar N (2006). Psychosocial issues following a natural disaster in a developing country: A qualitative longitudinal observational study. Int J Disaster Med.

[30] Kar N, Misra BN (2008). Mental health care following disasters.

[31] D’Netto TB (1963). Psychiatric first aid in community disasters, with special reference to Nuclear warfare. Indian J Psychiatry.

[32] Vijaykumar L, Thara R, John S, Chellappa S (2006). Psychosocial interventions after tsunami in Tamil Nadu, India. Int Rev Psychiatry.

[33] Vijayakumar L, Kannan GK, Ganesh Kumar B, Devarajan P (2006). Do all children need intervention after exposure to tsunami?. Int Rev Psychiatry.

[34] Rajkumar AP, Premkumar TS, Tharyan P (2008). Coping with the Asian tsunami: Perspectives from Tamil Nadu, India on the determinants of resilience in the face of adversity. Soc Sci Med.

[35] Shetty H (2006). Awakening the kundalini of humour. Indian J Psychiatry.

[36] Murthy RS, Isaac MK (1987). Mental health needs of Bhopal disaster victims and training of medical officers in mental health aspects. Indian J Med Res.

[37] Murthy RS, Kar N, Sekar K, Swain S, Mishra V, Daniel U Evaluation report on psychosocial care of survivors of supercyclone in Orissa.

[38] Rao K (2006). Lessons learnt in mental health and psychosocial care in India after disasters. Int Rev Psychiatry.

[39] Shethi BB, Sharma M, Trivedi JK, Singh H (1987). Psychiatric morbidity in patients attending clinics in gas affected areas in Bhopal. Indian J Med Res.

[40] Shetty H (1997). Marathawada earthquake. In: Proceedings of National workshop on Psychosocial consequences of disasters.

